# Multicultural Quality of Life Index in Relatives of People With Borderline Personality Disorder

**DOI:** 10.3389/fpsyt.2022.897692

**Published:** 2022-06-17

**Authors:** Jose Heliodoro Marco, Isabel Fernandez-Felipe, Joaquín García-Alandete, Sara Fonseca-Baeza, Rosa M. Baños, Azucena Garcia-Palacios, Sandra Pérez Rodríguez, Verónica Guillén

**Affiliations:** ^1^Department of Personality, Evaluation, and Psychological Treatments, University of Valencia, Valencia, Spain; ^2^CiberObn Pathophysiology of Obesity and Nutrition (CB06/03), Instituto de Salud Carlos III, Madrid, Spain; ^3^Department of Basic Psychology, Clinic and Psychobiology, Jaume I University, Castellón de la Plana, Spain

**Keywords:** quality of life, psychometric properties, relatives, borderline personality disorder, confirmatory factor analysis

## Abstract

**Purpose:**

The aim of the present study was to confirm the original factor structure of the *Multicultural Quality of Life Index* (MQLI) and analyze its psychometric properties in a sample of caregivers of people with borderline personality disorder (BPD).

**Methods:**

The MQLI was administered to 233 relatives of people with BPD. Participants completed the MQLI, the Depression, Anxiety and Stress Scale (DASS-21), and the Connor-Davidson Resilience Scale (CD-RISC).

**Results:**

Factor analysis of the relatives indicated that the MQLI generated a one-factor solution. The MQLI showed good internal consistency, ϖ = 0.91 [95% CI (0.90, 0.93)] and correlated significantly and positively with the CD-RISC (*r*_s_ = 0.576) and negatively with the DASS-21 (*r*_s_ = −0.583).

**Conclusion:**

Consistent with other studies, the MQLI demonstrated feasibility, strong internal consistency, and good convergent and discriminant validity, which means it is a psychometrically robust measure for the assessment of quality of life in relatives of people with BPD. Along with other validation studies, this measure will be a useful tool for assessing quality of life in relatives of people with mental disorders.

## Introduction

Quality of life (QoL) is becoming one of the key concepts in the healthcare system and social policies, and it is defined as individuals' perceptions of their position in life within a cultural context and their values in relation to their life goals, expectations, concerns, and norms (1). A study by Spitzer et al. (2) indicated that the main goal of the healthcare system is to improve patients' perceptions of their health in relation to their QoL, which is quite important when calculating the cost-effectiveness of treatments (3–5).

Borderline personality disorder (BPD) is associated with severe functional impairment, high use of healthcare resources, a worrisome percentage of suicide rates, and high comorbidity with other mental disorders (6). The characteristics of this disorder, such as emotional instability, impulsivity, fear of abandonment, inappropriate anger, and chronic feelings of emptiness, among others, may explain these negative consequences (6, 7). All these aspects could impact the QoL of individuals with BPD and their relatives. In terms of the influences of this disorder on people with BPD, it should be noted that relatives play an important role in its development, and that its symptomatology affects the family climate. Some studies (8, 9) indicate that relatives of people with BPD have high rates of burden, impaired wellbeing, high levels of psychological distress, and difficulties in emotion regulation, due to their family member's illness. Stress, lack of social and emotional support, economic hardship, and negative experiences produce emotional changes in relatives' QoL (10–12). Thus, it is important to assess and improve QoL in relatives with serious mental illness (e.g., BPD) in order to foster their own health and influence their patients' health by providing them with better care (13, 14).

The Multicultural Quality of Life Index (MQLI) is a self-report originally developed by Mezzich et al. (15). It was constructed to provide a measure of QoL based on the consensus of subject matter experts from several countries, subjective assessment of satisfaction with each domain, and the importance of each domain to each person. The MQLI is a 10-item questionnaire with a Likert-type response scale ranging from 1 (Poor) to 10 (Excellent) that assesses the following areas: physical and psychological wellbeing (e.g., feeling good about oneself), self-care and independent functioning (e.g., performing daily tasks), occupational and interpersonal functioning (e.g., performing one's job; relating well to family, friends, and groups), socioemotional support (e.g., having people to confide in), community and service support (e.g., safe, resourceful neighborhood, access to resources), personal and spiritual fulfillment (e.g., feeling of personal balance; feeling of faith), and overall perception of QoL (e.g., feelings of satisfaction and happiness in one's life).

The MQLI has been validated in different languages, including Spanish, English, Chinese, Korean, and Greek. The procedure for all these validations was carried out with exploratory factor analysis, except the Greek version, which used confirmatory factor analysis. Each validation confirmed the original factorial structure while maintaining the number of items. The Spanish version (15) used two samples of Spanish speakers, one with 60 psychiatric patients and the other with 20 health professionals, obtaining a Cronbach's alpha of 0.89 (in patients) and 0.97 (in health professionals). The sample in the English version (16) consisted of 124 psychiatric patients (α = 0.91) and 53 health professionals (α = 0.90). For the Chinese version (17), they studied a sample containing 124 psychiatric patients (α = 0.94) and 20 health care professionals (α = 0.95). The Korean version (18) used two adult samples, one with 100 psychiatric patients and the other with 30 health professionals, obtaining a combined Cronbach's alpha of 0.97. Finally, 884 Community-dwelling adults participated in the Greek version (19), which showed a Cronbach's alpha of 0.90. For discriminant validity, they used the Depression, Anxiety and Stress Scale (20).

As we can see in the aforementioned studies, the MQLI has been validated in different samples and settings, but research on its psychometric characteristics in relatives of people with mental disorders is scarce. The only published validation of the MQLI in a sample of relatives was carried out by Mundal et al. (21) with a sample of 128 relatives of children with attention deficit hyperactivity disorder (ADHD). They obtained good reliability, with a Cronbach's alpha of 0.73. In addition, the correlation between the two MQLI measures and the five-item World Health Organization Wellbeing Index (22) was high (*r* = 0.84).

Moreover, a construct related to QoL is resilience. It is a dynamic process that leads to successful individual adjustment in the face of adversity (23–27). Resilience has been positively associated with QoL, and the relationship between quality of life and resilience can occur in two ways. The first suggests that having higher QoL generates more adaptive coping strategies that result in greater resilience in the person. The second indicates that having greater resilience leads to more adaptive coping and, consequently, to higher QoL (28). To date, no studies have analyzed the relationship between resilience and QoL in relatives of people with BPD. Thus, confirming the relationship between these two variables would allow us to improve current interventions designed to help relatives of people with BPD.

In sum, the MQLI has been validated in numerous studies; however, it has never been validated in relatives of people with BPD. Taking into account that relatives of people with BPD present high levels of anxiety, depression, and burden (29), it is necessary to have reliable instruments to assess their QoL. Adequately assessing the QoL of these relatives could help to detect people at risk of developing psychological problems.

Therefore, the present study has two aims: (1) to analyze the psychometric characteristics and confirm the original factor structure of the MQLI in a sample of relatives of people with BPD; and (2) to study the evidence of the scale's convergent and discriminant validity by relating it to other measures: resilience and depression, anxiety, and stress.

## Materials and Methods

### Participants

The sample consisted of 233 relatives of patients with BPD who were receiving treatment at a Specialized Unit for Personality Disorders with four care facilities in the Valencian Community and one association of relatives of people with BPD in Spain. Recruitment was carried out from 2018 to 2021. The inclusion criteria were: (a) being a relative of a patient who met the criteria for BPD according to the Diagnostic and Statistical Manual of Mental Disorders (DSM-5) (30); (b) agreeing in writing to voluntarily participate in the study by signing the informed consent form. The exclusion criterion was the presence of a severe mental disorder in the relative (psychosis, schizophrenia, bipolar disorder, substance dependence, dementia, severe depression). This study was approved by the Ethics Committee of the University of Valencia with code: UV-INV_ETICA-1623849.

Regarding the gender of the participants, 67.4% (*n* = 157) were women, and 32.6% (*n* = 76) were men. Regarding the family role, 59.7% (*n* = 139) were mothers, 22.3% (*n* = 52) were fathers, 6% (*n* = 14) were son/daughters, 5.6% (*n* = 13) were partners, 3.4% (*n* = 8) were brothers or sisters, 1.3% (*n* = 3) were partners of the mother, 0.9% (*n* = 2) were uncles, and 0.4% (*n* = 1) was a grandfather. One participant did not report his/her family role. The mean age of the participants was 54.44 years (*SD* = 10.09).

Most of the relatives were married or living with a partner (56.6%; *n* = 132), 22.7% (*n* = 53) were single and 20.6% (*n* = 48) were separated, divorced, or widowed. As for the level of education, 8.2% (*n* = 19) had no studies, 22.3% (*n* = 52) had primary education, 29.6% (*n* = 69) had secondary education, and 39.9% (*n* = 93) had higher education.

### Instruments

#### The Multicultural Quality of Life Index

The Multicultural Quality of Life Index (MQLI) (15) has been extensively described in the introduction.

#### Depression, Anxiety, and Stress Scale

The Spanish version (31) of the Depression, Anxiety, and Stress Scale (20) has been used in this study.. It is a 21-item Self-report that assesses the severity of a range of problems common to depression, anxiety, and stress. It uses a Likert-type response scale ranging from 0 (it does not happen to me) to 3 (it happens to me a lot or most of the time). The Depression scale assesses dysphoria, hopelessness, and anhedonia, among others. The Anxiety scale assesses autonomic arousal, skeletal muscle effects, situational anxiety, and subjective experience of anxious affect. The Stress scale is sensitive to levels of chronic non-specific arousal. It assesses difficulty relaxing, nervous arousal, and being easily upset/agitated, irritable/over-reactive, and impatient. Higher values indicate more severe negative emotional symptoms. Regarding the internal consistency, Cronbach's alphas were excellent, ranging from 0.94 to 0.87. In this study, Cronbach's alphas ranged from 0.94 to 0.84. In the present study, the Depression, Anxiety, and Stress Scale (DASS-21) showed good internal consistency, ϖ = 0.95 [95% CI (0.94, 0.96)], which did not improve if an item was dropped.

#### The Connor–Davidson Resilience Scale

The Connor-Davidson Resilience Scale (32) is a 25-item self-report measure that assesses a broad range of resilience characteristics, including resilience, personal competence, tolerance of negative emotions, positive acceptance of change, personal control, and spirituality. It uses a Likert-type response scale ranging from 0 (not at all) to 4 (almost always). The Connor–Davidson Resilience Scale (CD-RISC) is a psychometrically sound measure of resilience that was designed to be used as an outcome measure. In the present study, the CD-RISC showed good internal consistency, ϖ = 0.90 [95% CI (0.87, 0.92)], which did not improve if an item was dropped.

### Procedure

Participants in the study were from two institutions located in Spain: (a) a Specialized Unit for Personality Disorders and (b) the Association for Family members of persons with BPD. The diagnostic interviews were conducted by six clinical psychologists with doctoral degrees and more than 10 years of experience in the assessment and treatment of BPD. Once the study had been explained to the family members, they were offered the opportunity to participate in the study. Interested family members signed the informed consent form, and the clinical psychologists carried out a clinical interview to verify that they met the inclusion and exclusion criteria. Then the participants filled in the assessment protocol: MQLI, DASS-21, and CD-RISC.

### Statistical Analyses

First, we analyzed the descriptive statistics (means and standard deviations), measures of data distribution (skewness and kurtosis), and internal consistency (McDonald's omega, ϖ) of the scales used in the present study and the MQLI items, as well as the item-rest correlations and the change in McDonald's ϖ of the MQLI if an item was dropped.

Second, we carried out a confirmatory factor analysis (CFA) to test the fit of the unidimensional model proposed for the MQLI to date. Because Mardia's coefficient was >5 (it was 8.9123) and the MQLI is an ordinal scale, robust (33) and Diagonally Weighted Least Squares (DWLS) (34) methods were used (35). The fit indices used were the Comparative Fit Index (CFI; values ≥0.90 indicate acceptable fit, and values ≥0.95 indicate good fit), the Tucker-Lewis Fit Index (TLI; values ≥0.90 indicate acceptable fit, and values ≥0.95 indicate good fit), the Root Mean Square Error of Approximation index (RMSEA; values lower than 0.080 indicate acceptable fit, and values <0.50 indicate good fit), and the Standardized Root Mean Square Residual index (SRMR; values <0.080 indicate acceptable fit, and values <0.050 indicate good fit) (36).

Third, to analyze the construct validity of the MQLI, the correlations (Spearman Spitzer s *rho, r*_*s*_) with both the CD-RISC (to test concurrent validity) and the DASS-21 (to test divergent validity) were analyzed.

All these statistical analyses were carried out with the JASP0.15 software (37).

## Results

The main result of this study was that the MQLI showed adequate psychometric properties, that is, a good internal consistency and both factorial and construct validity.

### Descriptive Statistics and Correlations

Table 1 shows the descriptive statistics, skewness, kurtosis, and internal consistency of the scales used in the present study in the whole sample and in the subsamples of men and women.

**Table 1:** Descriptive statistics and internal consistency of the scales used in the present study.

	**Scale**	***M*** **(*SD*)**	***Sk*** **(*SE* of *Sk*)**	***K*** **(*SE* of *K*)**	**ϖ (95% CI)**
Whole sample *N* = 233	MQLI	63.68 (15.82)	−0.222 (0.160)	−0.634 (0.319)	0.91 (0.90, 0.93)
	RS	2.55 (0.56)	−0.104 (0.187)	−0.240 (0.371)	0.90 (0.87, 0.92)
	DASS-21	2.54 (1.96)	0.931 (0.159)	0.369 (0.318)	0.95 (0.95, 0.96)
Men's subsample *N* = 76	MQLI	66.92 (15.58)	−0.335 (0.276)	−0.637 (0.545)	0.92 (0.98, 0.95)
	RS	2.53 (0.58)	−0.066 (0.314)	0.073 (0.618)	0.91 (0.87, 0.94)
	DASS-21	1.80 (1.66)	1.091 (0.276)	0.397 (0.545)	0.95 (0.93, 0.96)
Women's subsample *N* = 157	MQLI	62.09 (15.75)	−0.177 (0.195)	−0.596 (0.387)	0.91 (0.89, 0.93)
	RS	2.57 (0.55)	−0.122 (0.229)	–.388 (0.455)	0.89 (0.86, 0.92)
	DASS-21	2.90 (1.99)	0.867 (−194)	0.248 (0.385)	0.95 (0.94, 0.96)

*MQLI, multicultural quality of life index; RS, resilience scale; DASS-21, depression, anxiety and stress scale-21; Sk, skewness; SE, standard error; K, Kurtosis*.

Tables 2–4 show the descriptive statistics, skewness, and kurtosis of the MQLI items, the item-total correlations, and the MQLI's internal consistency if any item was dropped in the whole sample (Table 2) and in the subsamples of men (Table 3) and women (Table 4).

**Table 2:** Descriptive statistics, total-item correlations, and ϖ if item is dropped from the MQLI (whole sample).

**MQLI items**	***M*** **(*SD*)**	***Sk***	***K***	***r*** **_s_ total-item**	**ϖ if item dropped**
1. Physical wellbeing**/**Bienestar físico (sentirse con energía, sin dolores ni problemas físicos)	5.83 (2.11)	−0.133	−0.632	0.648	0.91
2. Psychological/emotional wellbeing/Bienestar psicológico/emocional (sentirse bien y satisfecho consigo mismo)	5.58 (2.11)	−0.081	−0.785	0.761	0.90
3. Self-care and independent functioning/Auto-cuidado y funcionamiento independiente (cuida bien de su persona, toma sus propias decisiones)	7.22 (1.92)	−0.596	−0.018	0.698	0.91
4. Occupational functioning/Funcionamiento ocupacional (capaz de realizar trabajo remunerado, tareas escolares, y tareas domésticas)	7.73 (2.06)	−1.013	−0.651	0.620	0.91
5. Interpersonal functioning/Funcionamiento interpersonal (capaz de responder y relacionarse bien con su familia, amigos y grupos)	7.66 (1.81)	−0.613	−0.424	0.699	0.91
6. Social emotional support/Apoyo social-emocional (disponibilidad de personas en quien puede confiar y de personas que le proporcionen ayuda y apoyo emocional)	6.75 (2.18)	−0.503	−0.307	0.667	0.91
7. Community and services support/Apoyo comunitario y de servicios (buen vecindario, disponibilidad de recursos financieros y de otros servicios)	6.17 (2.27)	−0.487	−0.260	0.634	0.91
8. Personal fulfillment/Plenitud personal (sentido de equilibrio personal, de autogobierno, de solidaridad, y de disfrute sexual y estético)	5.81 (2.20)	−0.192	−0.733	0.812	0.90
9. Spiritual fulfillment/Plenitud espiritual (experimentar una elevada filosofía de vida, religiosidad y trascendencia más allá de una vida)	5.39 (2.32)	−0.150	−0.692	0.533	0.91
10. Global Perception of Quality of Life/Percepción global de Calidad de vida (sentirse satisfecho y feliz con su vida en general)	5.77 (2.13)	−0.195	−0.657	0.757	0.90

*N = 233. Skewness Standard Error = 0.160; Kurtosis Standard Error = 0.320. MQLI ω = 0.91*.

**Table 3:** Descriptive statistics, correlations of the MQLI, and ϖ if item is dropped from the MQLI (men's subsample).

**MQLI items**	***M*** **(*SD*)**	***Sk***	***K***	***r*** **_s_ total-item**	**ϖ if item dropped**
1. Physical wellbeing/Bienestar físico (sentirse con energía, sin dolores ni problemas físicos)	6.12 (1.93)	−0.149	−0.721	0.675	0.91
2. Psychological/emotional wellbeing/Bienestar psicológico/emocional (sentirse bien y satisfecho consigo mismo)	6.01 (2.06)	−0.179	−0.738	0.785	0.91
3. Self-care and independent functioning/Auto-cuidado y funcionamiento independiente (cuida bien de su persona, toma sus propias decisiones)	7.54 (1.82)	−1.105	1.481	0.658	0.92
4. Occupational functioning/Funcionamiento ocupacional (capaz de realizar trabajo remunerado, tareas escolares, y tareas domésticas)	7.92 (1.85)	−0.848	−0.253	0.569	0.92
5. Interpersonal functioning/Funcionamiento interpersonal (capaz de responder y relacionarse bien con su familia, amigos y grupos)	7.79 (1.78)	−0.684	−0.197	0.700	0.91
6. Social emotional support/Apoyo social-emocional (disponibilidad de personas en quien puede confiar y de personas que le proporcionen ayuda y apoyo emocional)	6.86 (2.22)	−0.609	−0.240	0.753	0.91
7. Community services support/Apoyo comunitario y de servicios (buen vecindario, disponibilidad de recursos financieros y de otros servicios)	6.42 (2.39)	−0.590	−0.162	0.670	0.92
8. Personal fulfillment/Plenitud personal (sentido de equilibrio personal, de autogobierno, de solidaridad, y de disfrute sexual y estético)	6.17 (2.27)	−0.395	−0.735	0.792	0.91
9. Spiritual fulfillment/Plenitud espiritual (experimentar una elevada filosofía de vida, religiosidad y trascendencia más allá de una vida)	5.87 (2.20)	−0.179	−0.441	0.601	0.92
10. Global Perception of Quality of Life/Percepción global de Calidad de vida (sentirse satisfecho y feliz con su vida en general)	6.38 (2.05)	−0.306	−0.715	0.762	0.91

*N = 76. Skewness Standard Error = 0.276; Kurtosis Standard Error = 0.545. MQLI ω = 0.92*.

**Table 4:** Descriptive statistics, correlations of the MQLI, and ϖ if item is dropped from the MQLI (women's subsample).

**MQLI items**	***M*** **(*SD*)**	***Sk***	***K***	***r*** **_s_ total-item**	**ϖ if item dropped**
1. Physical wellbeing/Bienestar físico (sentirse con energía, sin dolores ni problemas físicos)	5.69 (2.18)	−0.086	−0.641	0.638	0.90
2. Psychological/emotional wellbeing/Bienestar psicológico/emocional (sentirse bien y satisfecho consigo mismo)	5.37 (2.11)	−0.028	−0.784	0.737	0.90
3. Self-care and independent functioning/Auto-cuidado y funcionamiento independiente (cuida bien de su persona, toma sus propias decisiones)	7.06 (1.96)	−0.387	−0.366	0.710	0.90
4. Occupational functioning/Funcionamiento ocupacional (capaz de realizar trabajo remunerado, tareas escolares, y tareas domésticas)	7.63 (2.16)	−1.032	0.743	0.652	0.90
5. Interpersonal functioning/Funcionamiento interpersonal (capaz de responder y relacionarse bien con su familia, amigos y grupos)	7.60 (1.83)	−0.585	−0.497	0.703	0.90
6. Social emotional support/Apoyo social-emocional (disponibilidad de personas en quien puede confiar y de personas que le proporcionen ayuda y apoyo emocional)	6.70 (2.16)	−0.457	−0.295	0.630	0.90
7. Community and services support/Apoyo comunitario y de servicios (buen vecindario, disponibilidad de recursos financieros y de otros servicios)	6.05 (2.21)	−0.464	−0.255	0.608	0.90
8. Personal fulfillment/Plenitud personal (sentido de equilibrio personal, de autogobierno, de solidaridad, y de disfrute sexual y estético)	5.63 (2.16)	−0.113	−0.645	0.814	0.89
9. Spiritual fulfillment/Plenitud espiritual (experimentar una elevada filosofía de vida, religiosidad y trascendencia más allá de una vida)	5.16 (2.35)	−0.112	−0.799	0.485	0.91
10. Global Perception of Quality of Life/Percepción global de Calidad de vida (sentirse satisfecho y feliz con su vida en general)	5.46 (2.10)	−0.152	−0.620	0.745	0.90

*N = 157. Skewness Standard Error = 0.195; Kurtosis Standard Error = 0.389. MQLI ω = 0.91*.

In the whole sample, data distribution was moderately and negatively skewed (negative skewness for the item 4 was >-1) and platykurtic. Positive kurtosis was found for Item 3 in the men's subsample and for Item 4 in the women's subsample.

In the whole sample and in the men's subsample, all the item-scale correlations were >0.50. In the women's subsample, the item-scale correlation for Item 9 was slightly below 0.50. In the present study, the MQLI showed good internal consistency, ϖ = 0.91 [95% CI (0.90, 0.93)], which did not improve if an item was dropped.

### Structural Validity

**Figure 1:**
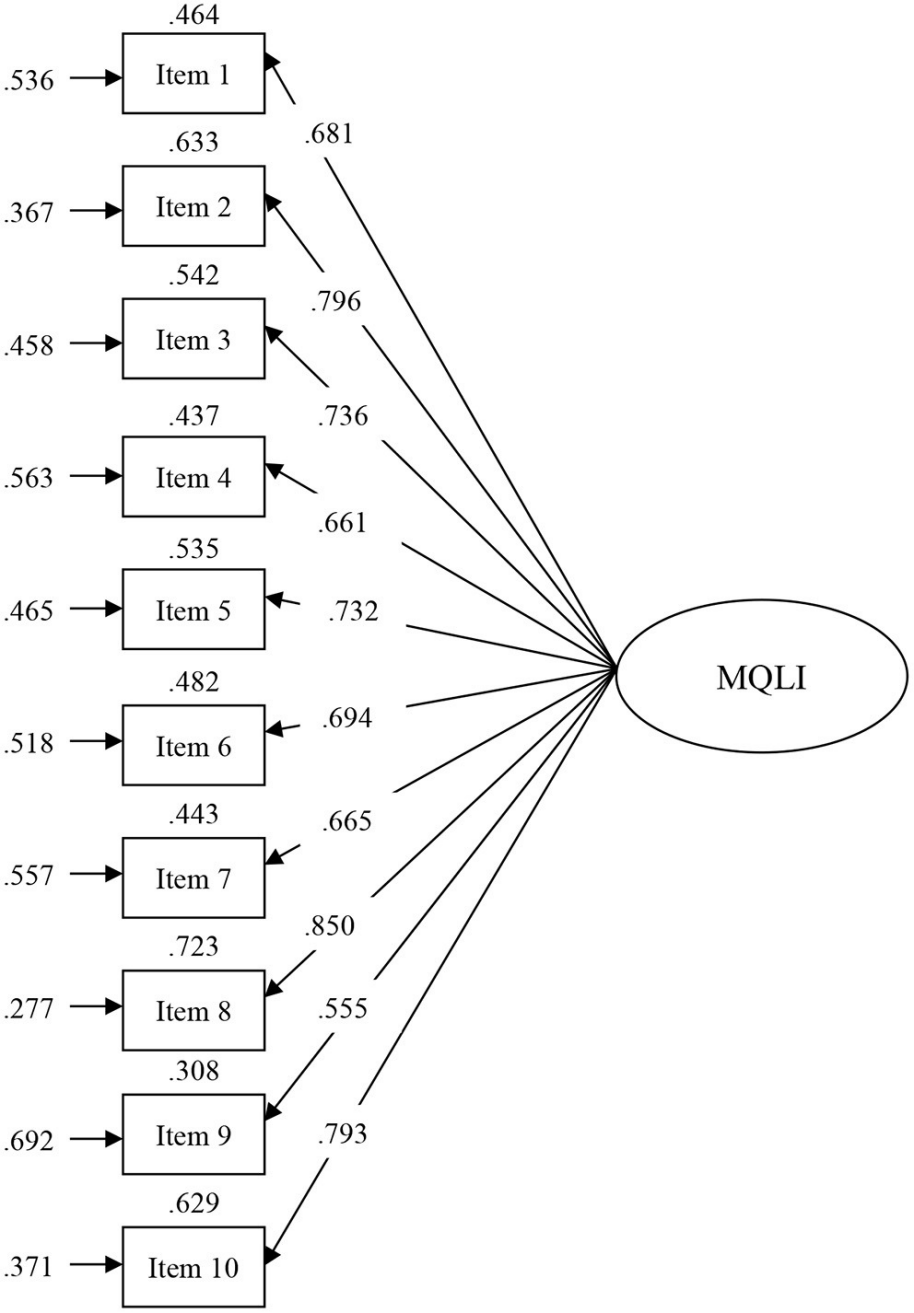
Model obtained in the present study for the MQLI. Values at the top of each rectangle are *R*^2^; values at the left of each rectangle are errors; parameter estimates and residual variances are standardized.

The unidimensional 10-item model for the MQLI showed a good fit: SB$\chi_{\left(35\right)}^{2}$ = 35.865, *P* = 0.428, CFI = 1.000, TLI = 1.000, RMSEA = 0.010, 95% CI [0.000, 0.049], SRMR = 0.057. The CFI index was >0.95, the RMSEA index was lower than 0.050, and the SRMR was <0.080 and close to 0.050. All parameters were significant at the 0.05 level (Figure 1).

### Construct Validity of the MQLI

The MQLI correlated significantly, *P* <0.001, and positively with the CD-RISC (*r*_*s*_ = 0.576) and negatively with the DASS-21 scale (*r*_*s*_ = −0.583), respectively. These strong correlations were in the expected direction, according to the construct assessed with each scale, Cohen (38).

## Discussion

The present study had the general objective of analyzing the psychometric properties of the MQLI in Spanish relatives of people with BPD. Specifically, the first aim was to study the internal structure of the MQLI, using a one-factor confirmatory model, and its internal consistency. The second aim was to study the evidence of the scale's convergent and discriminant validity by relating it to other measures: resilience and depression, anxiety, and stress.

Regarding the first aim, our results showed that the data had a good fit to a factorial model with one factor called QoL. This result confirms the original structure found by Mezzich et al. (15), and it also confirms the good psychometric properties of the MQLI. Moreover, the data from the present study confirm the results obtained with the QLI in numerous communities (15–19).

Regarding the second objective of the present study, the MQLI showed adequate convergent validity with measures of Resilience and psychopathology, as previous studies have shown (28). QoL was highly and positively associated with Resilience. This result suggests that these two constructs are strongly related, as indicated in previous studies that found that resilience was a significant predictor of QoL in individuals with schizophrenia, bipolar disorder, and healthy controls, such that higher resilience led to higher QoL (39, 40). Moreover, our results provide evidence of the divergent validity of the MQLI. The MQLI had a high and negative association with depression and a low and negative association with anxiety and stress. These results are similar to those from other studies (19) that found that QoL was negatively associated (*P* < 0.05) with severe depression, anxiety, and stress. We want to highlight that this is the first study to analyze the psychometric characteristics of the MQLI in relatives of people with BPD. Our results suggest that the MQLI is an adequate measure to evaluate QoL in this specific sample.

This study has several limitations. First, the sample, although sufficient to perform a CFA of the MQLI, is not large enough to study the invariance with respect to the gender and age of the participants. Thus, future studies should check whether the structural model of the MQLI is invariant for men and women and at different ages in a larger sample of Spanish participants, which, in turn, would make it possible to analyze gender and age-related differences. Moreover, our study does not include a test-retest analysis, and so future research should replicate our results in a longitudinal study and analyze test-retest reliability. Finally, we have used resilience as a measure to analyze convergent validity. Although resilience and QoL are related, they are two different constructs, and this is a clear limitation of our study. We propose adding another instrument that evaluates QoL to analyze convergent validity in future research.

Regarding clinical practice, this is a good instrument for the assessment of QoL in relatives of people with BPD, in order to easily and efficiently identify relatives who need psychological support and treatment for the problems they have with their loved ones. Thus, it is essential that patients receive adequate and continuous treatment and strong social support (14).

In sum, the present research provides support for the good psychometric properties and reliability of the MQLI in relatives of people with BPD, and the results suggest that the MQLI is an adequate measure to assess QoL.

## Data Availability Statement

The raw data supporting the conclusions of this article will be made available by the authors, without undue reservation.

## Ethics Statement

The studies involving human participants were reviewed and approved by Ethics Committee of the University of Valencia (Valencia, Spain) (UV-INV_ETICA-1623849). The patients/participants provided their written informed consent to participate in this study.

## Author Contributions

JM drafted the manuscript with important contributions from IF-F, AG-P, and VG. JM in collaboration with AG-P, VG, IF-F, RB, SF-B, SP, JG-A, and AG-P designed the study and participated in each of its phases. AG-P, VG, IF-F, SP, and JG-A participated in the review and revision of the manuscript and have approved the final manuscript to be published.

## Funding

This research received any specific grant from Consejeria de Innovación, Universidades, Ciencia y Sociedad Digital: Subvenciones para Grupos de Investigación Consolidables-AICO/2021 Ref: 20210862; and form Ministry of Education, Culture and Sport (FPU) by means of an FPU grant awarded to IF-F and SF-B (FPU17/04210; FPU15/07177).

## Conflict of Interest

The authors declare that the research was conducted in the absence of any commercial or financial relationships that could be construed as a potential conflict of interest.

## Publisher's Note

All claims expressed in this article are solely those of the authors and do not necessarily represent those of their affiliated organizations, or those of the publisher, the editors and the reviewers. Any product that may be evaluated in this article, or claim that may be made by its manufacturer, is not guaranteed or endorsed by the publisher.
